# Response to Treatment with an Anti-Interleukin-6 Receptor Antibody (Tocilizumab) in a Patient with Hemophagocytic Syndrome Secondary to Immune Checkpoint Inhibitors

**DOI:** 10.1155/2021/6631859

**Published:** 2021-02-12

**Authors:** Alejandro Olivares-Hernández, Luis Figuero-Pérez, María A. Amores Martín, Lorena Bellido Hernández, Laura Mezquita, María del Rosario Vidal Tocino, Félix López Cadenas, Felipe Gómez-Caminero López, Roberto A. Escala-Cornejo, Juan Jesús Cruz Hernández

**Affiliations:** ^1^Department of Medical Oncology, University Healthcare Complex of Salamanca, Paseo de San Vicente, 182, CP 37007 Salamanca, Spain; ^2^Instituto de Investigación Biomédica de Salamanca (IBSAL), Paseo de San Vicente, 58-182, CP 37007 Salamanca, Spain; ^3^Department of Medical Oncology, Hospital Clinic, Carrer de Villarroel, 170, CP 08036 Barcelona, Spain; ^4^Hematology Department, University Healthcare Complex of Salamanca, Paseo de San Vicente, 182, CP 37007 Salamanca, Spain; ^5^Nuclear Medicine Department, University Healthcare Complex of Salamanca, Paseo de San Vicente, 182, CP 37007 Salamanca, Spain; ^6^Department of Medical Oncology, Ávila Healthcare Complex, Avenida Rey Juan Carlos I, CP 05004 Ávila, Spain

## Abstract

*Background*. Immunotherapy represents one of the fundamental treatments in the management of some types of cancer, especially malignant melanoma. Toxicity derived from increased immune system activity can manifest in multiple organs and systems. We present a case of hematological toxicity, manifested as hemophagocytic syndrome (HPS), which was successfully treated with an anti-interleukin-6 antibody (tocilizumab). *Case Report*. This case presents a 75-year-old woman diagnosed with metastatic choroidal melanoma, refractory to several lines of treatment. After the failure of the previous lines, ipilimumab was started. After the third dose, she developed grade 2 thrombocytopenia and anemia accompanied by elevated levels of ferritin, triglycerides, and decreased fibrinogen. Hemophagocytosis was observed in the bone marrow biopsy, and a PET-CT showed splenomegaly with increased metabolism. Treatment was based on high doses of corticosteroids and tocilizumab. Four days after the start of treatment, progressive clinical and analytical improvement was observed, achieving total remission of the condition. *Discussion*. HPS induced by immunotherapy is due to an immunorelated cytokine storm syndrome (CSS). The administration of the anti-interleukin-6 receptor antibody drug acted on this cytokine cascade, leading to stabilization and subsequent remission. For this reason, the use of tocilizumab should be part of the immunotherapy-induced HPS treatment algorithm.

## 1. Background

Hemophagocytic syndrome (HPS) is a rare clinical entity characterized by hyperactivity and irregularity in the activation of the immune system induced by response to a specific trigger. This pathological immune activation manifests as signs and symptoms of excessive inflammation due to dysfunction of the natural killer (NK) cells, which leads to the overstimulation, hyperproliferation, and ectopic migration of T1 cells [1]. The incidence is estimated at 1.2 per million cases per year. However, these values may be underestimated due to low diagnostic suspicion in most cases [2].

HPS is not a single disease but a syndrome associated with a wide variety of underlying causes that lead to a characteristic inflammatory phenotype [3]. The triggering causes can be classified as primary in the case of a mutation of the FLH gene or as secondary when they are due to infectious, neoplastic processes or other immunodeficiencies; in general, the secondary causes are observed most frequently [4]. The most common symptoms are fever and splenomegaly, appearing in about 75% of patients at diagnosis, followed by liver failure, sepsis, Kawasaki disease, and neurological abnormalities. Diagnosis is based on a scale of eight diagnostic criteria, of which the patient must meet at least five: (1) fever (>38°C); (2) splenomegaly; (3) cytopenias that affect at least two series; (4) hypertriglyceridemia (>265 mg/dL) and/or low fibrinogen levels (<150 mg/dL); (5) hemophagocytosis in the bone marrow, spleen, lymph nodes, or liver; (6) low or no activity of the NK cells; (7) high levels of ferritin (>500 ng/mL); and (8) high levels of soluble CD25 [5].

Inhibition at the checkpoints of the immune system is currently one of the cornerstones of cancer treatment in a wide variety of tumors, including melanoma [6]. Current treatments for B-RAF wild-type melanoma are based on two blocking points: (1) inhibition of the cytotoxic T lymphocyte antigen 4 (CTLA-4) receptor and (2) inhibition of the programmed cell death protein 1 (PD-1) receptor [7]. Monoclonal antibodies directed towards CTLA-4 inhibition, such as ipilimumab, and anti-PD-1, such as nivolumab, have changed the treatment and natural evolution of metastatic melanoma. The immune-related adverse events (irAEs) from these treatments originate from excessive immune activation and can affect any system or body function [8]. Hematological toxicity appears in less than 1% of cases, with thrombocytopenia being the most frequently described [9]. The management of irAE is very complex, making the use of corticosteroids and immunosuppressive treatments the mainstay. [10] We present a case of a woman diagnosed with metastatic choroidal melanoma who developed HPS secondary to treatment with ipilimumab.

## 2. Case Report

A 75-year-old woman diagnosed with choroidal melanoma in 1989 was treated by enucleation without adjuvant treatment. After 22 years of disease-free survival (DFS), in 2011, a CT scan revealed liver involvement, which was confirmed by biopsy and indicated the recurrence of the B-RAF wild-type melanoma. She began treatment with dacarbazine (DTIC), which followed by nivolumab in 2016 due to tumor progression. With nivolumab, she maintained a progression-free interval (PFI) of three years. When new liver progression appeared, a third line of treatment with ipilimumab (3 mg/kg) was started, and she received three cycles.

**Figure 1:**
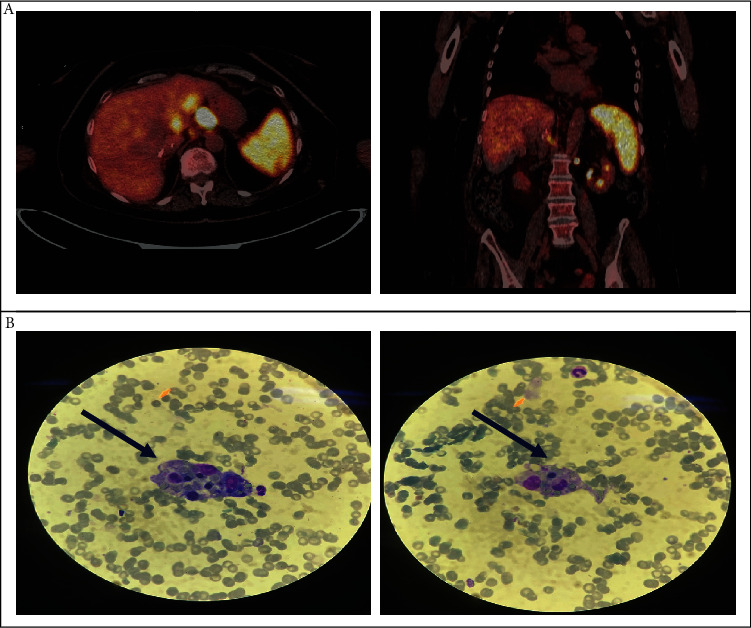
Pretreatment: (a) PET-CT: splenomegaly and increased metabolism in the spleen are observed at diagnosis; (b) bone marrow biopsy: macrophage phagocytosing erythrocytes and neutrophils (hemophagocyte).

Prior to the start of a fourth cycle of treatment with ipilimumab, grade 2 thrombocytopenia (platelets: 64,000/*μ*L) was observed, suggesting hematological toxicity associated with the immunotherapy treatment. Due to this suspicion, the treatment with anti-CTLA-4 was interrupted, and methylprednisolone was started at a dose of 1 mg/kg. Despite the corticosteroid treatment, no clinical analytical improvement was observed; therefore, the patient was admitted to the hospital admission for study and determination of the appropriate treatment. During admission, a PET-CT was performed, in which a partial response was observed in the liver. Splenomegaly was also observed with a diffuse increase in the metabolic activity of the entire splenic parenchyma. Suspecting primary splenic B lymphoma, a bone marrow biopsy was performed, and abundant macrophages with compatible characteristics of hemophagocytosis were observed (Figure 1).

High levels of ferritin (>85,000 ng/mL), hypertriglyceridemia (maximum value of 389 mg/dL), low levels of fibrinogen (147 mg/dL), and normochromic normocytic anemia (9.8 g/dL) were observed in the blood samples requested during admission. Furthermore, the patient presented a fever of up to 38.5°C without elevation of acute phase reactants, such as c-reactive protein (CRP). Based on all of these signs and symptoms, HPS was confirmed, and other possible causes were ruled out, including infections (negative viral serologies, as well as negative urine and blood cultures, were collected), autoimmune diseases, hematological malignancies, and bone marrow infiltration.

The patient met six of the eight diagnostic criteria for HPS. After ruling out other causes, secondary HPS was attributed to an immunomediated adverse effect secondary to treatment with the anti-CTLA-4, ipilimumab. Despite corticosteroid treatment (maximum doses of 2 mg/kg), no improvement was observed, and it was decided to start treatment with an anti-interleukin-6 receptor antibody (tocilizumab) at a dose of 8 mg/kg every eight hours for four doses [11]. Prior to the tocilizumab regimen, the IL-2 value was determined to demonstrate the existence of the cytokine storm secondary to HPS. The IL-2 value was 1734 U/mL (158-623 U/mL). After the fourth dose of tocilizumab, there was clinical improvement without fever and analytical improvement with progressive normalization of the levels of hemoglobin, platelets, ferritin, fibrinogen, and triglycerides (Table 1).

**Figure 2:**
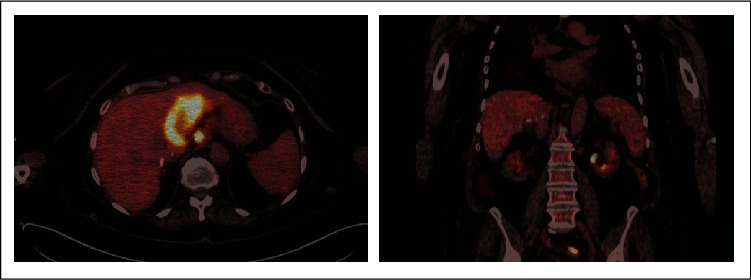
Posttreatment. PET-CT: significant decrease in the metabolism in the spleen, after 4 doses of tocilizumab.

Once the clinical process was corrected, a new bone marrow biopsy was performed, and no signs compatible with hemophagocytosis were found. In addition, a PET-CT was performed (Figure 2), in which an absence of splenic metabolic uptake was observed. For these reasons, the patient was discharged from the hospital and began outpatient follow-ups with the hematology and medical oncology departments.

## 3. Discussion

Immune-related adverse effects are more frequently observed with the use of anti-CTLA-4 (40%) than with anti-PD-1 or anti-PD-L1. The most common irAEs are asthenia, anorexia, and fever, appearing in up to 40-50% of cases. Hematologic irAEs are poorly described in the literature due to their low incidence (0.5%) [9].

It has been described that, in a small group of patients, treatment with immune checkpoint inhibitors could induce a cascade of cytokines, leading to the development of HPS. The cases reported in the literature suggest that HPS secondary to immunotherapy has been observed with the use of modified T cells (such as modified chimeric antigen receptor T (CAR-T) cells), blinatumomab (monoclonal antibody directed against CD3 and CD19) [12, 13], and immune checkpoint inhibitors.

Recently, the use of the anti-interleukin-6 receptor antibody (tocilizumab) has been reported and approved for the treatment of HPS secondary to the use of CAR-T cells and blinatumomab. However, in patients treated with immune checkpoint inhibitors, such as anti-CTLA-4 or anti-PD-1/PD-L1, tocilizumab is not typically used for HPS. In published cases of HPS as an irAE, immunomodulators have been used, such as etoposide [14], mycophenolic acid [15], and tacrolimus [16], and are always associated with corticosteroids in the interruption of immune treatment.

In the case report, the use of tocilizumab was a novel treatment in the field of immunotoxicity secondary to the anti-CTLA-4, introducing new hope into the treatment of this type of patient, with excellent results. In our center, the use of tocilizumab was based on the study carried out by Dupré et al., in which the possible efficacy of tocilizumab in the treatment of HPS secondary to immune checkpoint inhibitors was postulated [17]. Our patient presented clinical improvement with the absence of fever and normalization of laboratory levels when four doses of tocilizumab were administered. The benefit of the treatment and the short hospital stay highlights the effectiveness and the role that anti-interleukin-6 receptor antibodies could have in the treatment of HPS induced by immune checkpoint inhibitors. The development of a massive cytokine storm is the main determining factor in HPS secondary to treatment with immune checkpoint inhibitors. For this reason, the decrease in endogenous levels of interleukin-6 using tocilizumab could mitigate the inappropriate activation of macrophages and improve the clinical situation.

The treatment of any moderate or high grade irAEs, not only HPS, is based on the use of corticosteroids at high doses (1-2 mg/kg) [18]. Taking into account the current results on the use of the anti-interleukin-6 receptor antibody, we suggest that drugs, such as tocilizumab, could have a beneficial use in severe cases of irAEs, as well as in patients for whom the use of corticotherapy would be limited, such as those with type 1 diabetes mellitus secondary to immunotherapy [19].

In conclusion, our case and the review of the literature suggest that, at present, the anti-interleukin-6 antibodies may be an appropriate treatment option for HPS induced by immune checkpoint inhibitors. Likewise, more studies are needed to determine the validity of these treatments before being recommended in the clinical practice.

## Figures and Tables

**Table 1:** Evolution of analytical values during the admission of the patient.

Analytical values	HPS criteria	At time of admission	Baseline steroid therapy	Baseline tocilizumab	After 48 h of tocilizumab	At time of discharge
Hemoglobin (g/dL)	Yes	13.5	11.2	9.6	11.6	12.1
Platelets (/*μ*L)	Yes	64.000	45.000	48.000	74.000	113.000
Ferritin (ng/mL)	Yes	>74.150	>74.150	>89.918	12.019	1978
Fibrinogen (mg/dL)	Yes	147	164	170	—	—
Triglycerides (mg/dL)	Yes	271	327	265	234	172
Bone marrow findings	Yes	—	—	Yes	—	No

– Not available.

## Data Availability

The data provided for the case report can be found in the clinical record database of the University Hospital of Salamanca.
